# Development of Biodegradable and Biobased Poly(glycerol levulinate-*co*-glycerol malonate) Copolyesters with Controlled Degradation

**DOI:** 10.1021/acs.macromol.5c00383

**Published:** 2025-09-10

**Authors:** Huru Rabia Gulec, Zaid Kareem, Mete Karaboyun, Ersan Eyiler

**Affiliations:** Department of Chemical Engineering, 37506 Cukurova University, Adana 01950, Turkey

## Abstract

Fully biobased oligomers were synthesized from glycerol, levulinic acid, and malonic acid via melt polycondensation. Itaconic acid with a carbon–carbon double bond was incorporated to enable cross-linking using dicumyl peroxide. The effect of the monomer molar ratio on material properties was investigated to understand the structure–property relationships. The addition of malonic acid, acting as both a monomer and a secondary cross-linker, was found to tune the glass-transition temperature, thermal stability, and degradation behavior of the cross-linked random copolyesters. Thermogravimetric analysis (TGA) revealed that malonic acid significantly improved the thermal stability, increasing it by up to 7.6% compared to the neat polymer. The cross-linked copolyesters exhibited excellent degradation profiles, making them suitable for biomedical applications where controlled degradation is essential. Additionally, they demonstrated outstanding shape memory properties, with a nearly 100% shape recovery, offering further potential for biomedical device fabrication.

## Graphical abstract

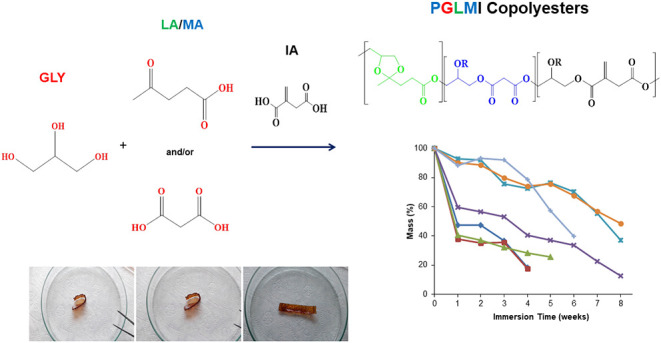

## Introduction

Degradable polymers are of great interest for biomedical applications, where controlled degradation is necessary, such as in drug delivery systems, sutures, stents, and implants.^1^ The ability to design polymers that degrade in a controlled manner allows for the development of temporary medical devices that do not require removal after they have performed their intended function.^2,3^ Recent advancements in biobased polymers have emphasized the importance of using renewable resources to reduce environmental impact and reliance on fossil fuels.^4^ Biobased polymers, derived from biomass, offer the potential benefit of biodegradability, making them ideal candidates for applications where both biocompatibility and environmental sustainability are essential. With the growing limitations of oil reserves, dependency on fossil fuels, and the environmental issues associated with petroleum-based plastics, interest in biobased polymers has surged.^5^ According to the World Economic Forum, biobased plastics for a circular economy was ranked as the top topic among the top 10 emerging technologies in 2019.^6^ Biomass offers the potential to provide the same chemical building blocks from fossil resources, which are essential for producing high-performance materials.^7^ Meanwhile, as energy, resources, and serious environmental problems are profoundly affecting our lives today, many scientists have drawn attention to the importance of utilizing renewable resources to reduce dependence on fossil fuels.^5,8,9^ Consequently, degradable polymers developed from biomass and bioproducts are gaining significance. Recent studies have highlighted the development of biobased degradable polymers with enhanced properties.

Several diacids and diols, such as 1,3-propanediol (PDO) and itaconic acid (IA), are produced industrially through fermentation.^10–12^ Among them, levulinic acid (LA), a five-carbon keto acid monomer, can be derived from six-carbon sugar carbohydrates found in starch or ligno-cellulosic biomass.^13^ Additionally, this biomonomer, which can be easily produced from abundantly available cellulose, has the potential to enhance properties through copolymerization with other monomers. It is listed as one of the 12 promising, value-added chemical building blocks derived from sugar by the U.S. Department of Energy.^10^ LA and its derivatives have been used as starting materials to develop polymers such as poly­(5-hydroxylevulinic acid) and poly­(5-hydroxylevulinic acid-*co*-L-lactic acid) polymers.^14,15^ However, while studies on the ketalization and esterification with certain alcohol monomers have been conducted, their success has been limited, depending on the catalysts used, and the direct polymerization of LA is less common. Amarasekara and his colleagues are the most active researchers in this field, although their work has remained at a certain level.^16,17^ In 2017, Okorie et al. synthesized polyacetals by reacting LA with pentaerythritol using Brönsted and Lewis acid catalysts.^18^ Sb_2_O_3_ (a Lewis acid) produced polymers with molecular weights up to 19 kDa, a 98% yield, and a thermal stability up to 320 °C. Similarly, Hawkins et al. reacted glycerol with LA using three catalysts, where Sb_2_O_3_ gave the highest polymerization (9.7 units) and stability up to 275 °C.^17^

Malonic acid (MA), on the other hand, has not been extensively studied for polymer applications but is a monomer suitable for the development of linear copolymers. It is a highly valuable chemical used in electronics, flavor and fragrance, polymer cross-linking, and pharmaceuticals.^19^ MA acts as an effective cross-linker, enhancing the flexibility of the polymers. With a molecular weight of 104 g/mol, it is a water-soluble dicarboxylic acid available as white crystals, commonly used as a precursor for polyesters and alkyd resins.^20,21^ Naturally found in high concentrations in beets, MA is commercially produced through a microbial process using sugar and water, resulting in relatively low production costs. Both LA and MA offer unique properties that can significantly contribute to the advancement of polymer technologies and serve as potential raw materials for biobased polyesters.

While our previous work reported on fully biobased block copolyesters incorporating glycerol, 1,3-propanediol, levulinic acid, malonic acid, and itaconic acid, which exhibited thermal degradation temperatures up to 353 °C and showed around 71% mass remaining after 8 weeks of in vitro degradation, the current study explores random copolyesters synthesized solely from glycerol, levulinic acid, malonic acid, and itaconic acid.^22^ The exclusion of 1,3-propanediol and adoption of a random copolymer architecture provide distinct thermal, mechanical, and degradation properties suitable for biomedical applications such as shape memory stents. In this study, fully biobased oligomers using glycerol, levulinic acid, malonic acid, and itaconic acid were synthesized via melt polycondensation techniques. This work represents one of the first attempts to utilize levulinic acid and malonic acid in combination with other renewable monomers for the development of biobased copolyesters specifically designed for potential biomedical applications where controlled degradation and optimal material performance are essential. The effect of the monomer molar ratio on material properties was investigated to understand structure–property relationships. The synthesized copolyesters were characterized for their chemical properties through Fourier transform infrared spectroscopy (FTIR), and thermal properties were analyzed with differential scanning calorimetry (DSC) and thermogravimetric analysis (TGA). Additionally, in vitro degradation tests were conducted on the cross-linked random copolyester films.

## Experimental Section

### Materials

Levulinic acid (LA, 98%), malonic acid (MA, 99%), glycerol (GLY, ≥99.5%), itaconic acid (IA, 99 + %), antimony­(III) oxide (Sb_2_O_3_, 99%), 4-methoxyphenol (HQ, 99%), dicumyl peroxide (DCP, 98%), tetrahydrofuran (THF, ≥99.9%), chloroform (CHCl_3_, ≥99.4%), and other solvents were purchased from Sigma-Aldrich. All reagents and solvents were used as received without further purification. Although Sb_2_O_3_, HQ, and DCP are not biobased, they were used in minimal amounts due to their well-established effectiveness in catalysis, stabilization, and cross-linking, respectively. Efforts to identify greener and less toxic alternatives are ongoing.

### Synthesis of Oligomers

A series of poly­(glycerol levulinate-*co*-glycerol malonate-*co*-glycerol itaconate) (PGLMI) copolyesters were synthesized by a two-step melt polycondensation. Reactions took place in a 100 mL round-bottom flask equipped with a distillation apparatus and nitrogen purge to remove water and volatile byproducts. The molar ratio of diol to diacid was maintained at 1.1:1, with the itaconic acid content fixed at 10 mol % relative to the total acids, based on previous reports indicating that a slight excess of diol enhances molecular weight in melt polycondensation, while 10 mol % IA ensures efficient cross-linking without compromising flexibility.^23,24^ In a typical synthesis, LA (0.045 mol), glycerol (0.11 mol), MA (0.045 mol), IA (0.01 mol), and 0.5 wt % HQ (based on total reactants) were combined and stirred under nitrogen to suppress oxidation. The mixture was heated to 135 °C and held for 3 h for oligomerization. Next, the Sb_2_O_3_ catalyst (0.1 mol % relative to acid units) was introduced, and the reaction temperature was increased to 210 °C under vacuum (∼7 mbar) for 3 h to drive polycondensation. The resulting viscous, yellowish resin was used directly for further analysis and cross-linking without purification. Seven copolymers were prepared by varying the LA/MA feed ratios: 100/0, 80/20, 60/40, 50/50, 40/60, 20/80, and 0/100. The copolymers were labeled as “PGLxMyI”, where *x* and *y* denote the molar fraction of levulinate and malonate, respectively. The homopolymers PGLI and PGMI correspond to PGL_100_M_0_I and PGL_0_M_100_I, respectively. The overall synthesis pathway is illustrated in Scheme 1, while detailed mechanisms for the key reactions—Fischer esterification and ketalization between glycerol and levulinic acid—are provided in Scheme S1 for better mechanistic understanding.

**Scheme 1:**
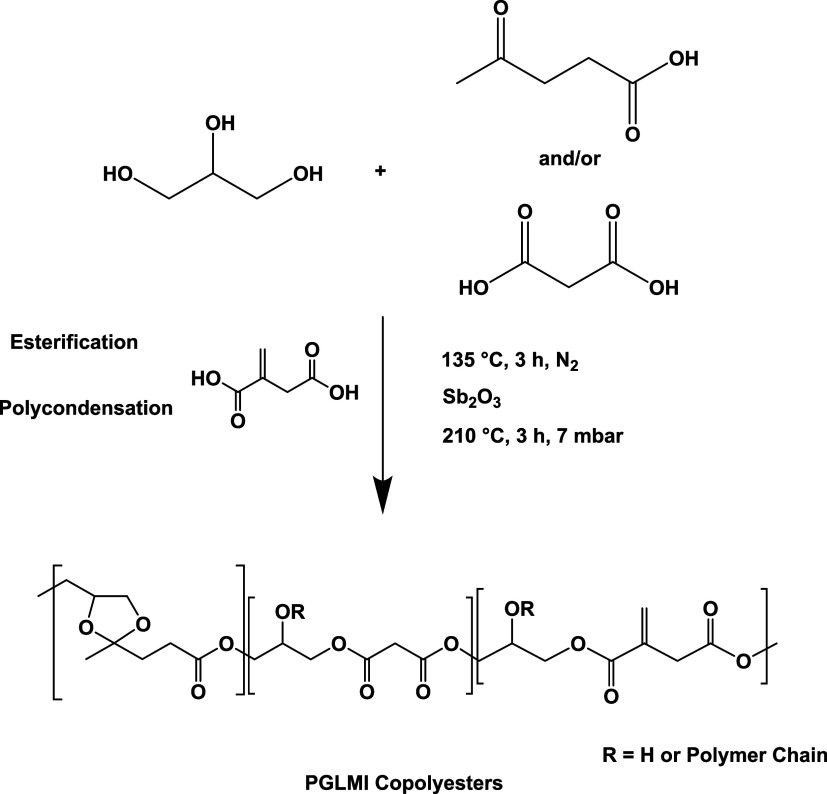
Proposed Reaction Process for the Synthesis of PGLMI Oligomers

### Preparation of Cross-Linked Films

For film fabrication, PGLMI oligomers were mixed with 0.5 wt % dicumyl peroxide (DCP) as a free-radical initiator and heated to 50 °C while stirring magnetically for 5 min to reduce viscosity and ensure homogeneity. The resulting mixture was then cast into siliconized Teflon molds (∼70 mm diameter). Samples were cured in an air oven at 160 °C for 24 h. After curing, the films were carefully removed from the molds.

### Characterization

Nuclear magnetic resonance (^1^H and ^13^C NMR) spectra were recorded on a Bruker Ultrashield Plus Biospin Avance III 400 MHz spectrometer, using tetramethylsilane (TMS) as an internal reference and CDCl_3_ as a solvent. Fourier transform infrared (FTIR) spectra were collected by using a PerkinElmer RX-1 spectrometer over the 400–4000 cm^–1^ range with 64 scans per sample. Molecular weights and dispersity indices were determined by gel permeation chromatography (GPC) on an Agilent 1200 system, equipped with a refractive index detector and PLgel Mixed-D (5 μm) and Mixed-E (3 μm) columns. Tetrahydrofuran stabilized with BHT and TEA served as the eluent at 1 mL/min. PMMA standards were used for calibration, and samples were prepared at a 1 mg/mL concentration.

Differential scanning calorimetry (DSC) was performed on a Mettler Toledo DSC 3. Samples were heated from room temperature to 160 °C at 10 °C/min and held for 3 min to erase thermal history, then cooled to −60 °C and reheated to 160 °C at the same rate. The glass-transition temperature (Tg) was determined by using the midpoint method from the second heating cycle. Thermogravimetric analysis (TGA) was conducted on a Mettler Toledo TGA/DSC 3+ instrument under a nitrogen flow (40 mL/min), heating samples from 25 to 600 °C at 10 °C/min. Dynamic mechanical analysis (DMA) was carried out using a PerkinElmer DMA 8000 in tensile mode on samples measuring 10 × 5 × 0.6 mm^3^, heated from −50 to 50 °C at 3 °C/min with a frequency of 1 Hz.

Shape memory behavior was evaluated by a bending test: samples (50 × 5 × 0.6 mm^3^) were immersed in 20 °C water for 3 min, bent around a 7 mm diameter glass rod into a “*U*” shape, held for 5 s, and then frozen at −20 °C for 30 min. Bending angles were recorded after 3 min at room temperature and again after reheating in 20 °C water for 2 min.

Gel content, swelling ratio, cross-link density, and average molecular weight between cross-links (Mc) were calculated based on swelling experiments (details in the Supporting Information). Cross-linked film samples (10 × 5 × 0.6 mm^3^) were weighed (initial weight, *w_i_*), soaked in THF for 24 h to remove soluble components, and then dried under vacuum to obtain the dry weight (*w*_d_). The gel content and swelling ratio were calculated based on weight changes, and the cross-link density and Mc were determined using the Flory–Rehner theory.

In vitro degradation of the cross-linked film samples was assessed by immersing cured films in phosphate-buffered saline (PBS, pH 7.4) at 37 °C for up to 8 weeks. Samples (10 × 5 × 0.6 mm^3^) were weighed (w_i_) before immersion in 10 mL of PBS with stirring. Weekly, samples were removed, rinsed with deionized water, dried under a vacuum, and weighed (*w_t_*). The pH of PBS was recorded immediately after the removal of samples. The weight change was determined by

1 $$w\mathrm{eight change}\;\left(\%\right)=\frac{w_{t}}{w_{i}}\times 100$$

All degradation tests were performed in triplicate.

## Results and Discussion

### Chemical Structure of Oligomers and Cross-Linked Copolyester Films

#### NMR

Different PGLMI oligomers were produced by following a two-step melt polycondensation method (Scheme 1). The oligomers appear as a viscous, transparent, yellowish liquid, and they were fully soluble in THF and CHCl_3_, indicating no cross-linking via carbon–carbon double bonds. After synthesis, the chemical structure and composition of the synthesized PGLMI oligomers were characterized by ^1^H and ^13^C NMR, respectively, as illustrated in Figures 1 and S1. The CH_3_ group of the unreacted LA can be observed as a singlet at 2.21 ppm, whereas two small triplets at 2.60 and 2.79 ppm correspond to the methylene protons of LA. The absorption at 2.44 ppm can be assigned to −CH_2_– groups from ketal-ester. Malonic acid derivatives are prone to decarboxylation (Scheme S2), a reaction often involving ester groups. The 2.1 ppm peak, corresponding to the acetate methyl group (−COOCH_3_), is typically seen in such decarboxylation reactions. As the malonic acid (MA) content increased, the intensity of the 2.1 ppm peak also increased, while the intensity of the 3.45 ppm peak, which corresponds to the methylene protons (−CH_2_) of the malonic acid diester, remained constant. The 3.45 ppm peak is deshielded due to the adjacent ester groups. The peaks between 3.58 and 4.43 ppm were ascribed to glycerol and the 1,3-dioxolane ring. The chemical shifts at 6.39, 5.79, and 3.41 ppm correspond to −CO–C­(=CH_2_)–CH_2_– from itaconic acid, demonstrating the introduction of pendant alkene groups to the PGLMI chains. The molar composition of PGLMI was determined by calculating the relative areas of the methyl peaks at 1.37 and 2.1 ppm related to levulinic acid and malonic acid, and the monomer ratios in the product were in good agreement with the feed ratios (Table 1).

**Figure 1:**
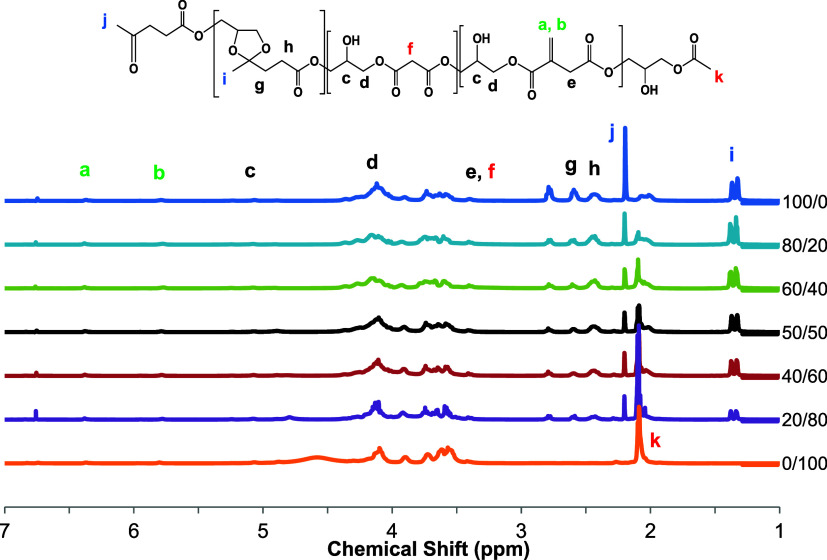
^1^H NMR spectra for all uncured PGLMI oligomers.

**Table 1:** Composition and Molecular Weight of Uncured PGLMI Oligomers

sample	feed GL/GM ratio (mol %)	^1^H NMR GL/GM ratio (mol %)	*M_n_* (g/mol)	*M*_w_ (g/mol)	*Đ*
PGLI	100/0	100/0	590.32	786.7	1.33
PGL_80_M_20_I	80/20	78/22	647.28	884.53	1.37
PGL_60_M_40_I	60/40	57/43	617.5	798.52	1.29
PGL_50_M_50_I	50/50	50/50	507.46	625.67	1.23
PGL_40_M_60_I	40/60	40/60	468.6	541.58	1.16
PGL_20_M_80_I	20/80	20/80	242.41	343.29	1.42
PGMI	0/100	0/100	511.95	536.09	1.08

The ^13^C NMR spectrum of the repeating unit of the PGLMI oligomers revealed distinct signals at 109.79 and 110.08 ppm (C-2), 73.31 and 74.23 ppm (C-4), and 66.31 ppm (C-5) corresponding to the carbons in the 1,3-dioxolane ring.^17^ The methyl group attached to C-2 of the 1,3-dioxolane ring appeared as two equal-intensity peaks at 23.67 and 24.79 ppm. Additionally, the ester carbonyl group in the repeating unit was observed at 173.09 ppm, which further confirmed the ester–ketal structure of the oligomer. The esterified glycerol unit was identified by three distinct ^13^C peaks at 63.43, 69.91, and 65.26 ppm, corresponding to C-1, C-2, and C-3 of the glycerol backbone, respectively.

The oligomeric products with low molecular weights (*M_n_*) between 242.41 and 647.28 g/mol were obtained, and their low dispersity (*Đ*) values were in the range of 1.08–1.42, which indicates a uniform distribution of molecular weights (Table 1). This is attributed to the polymerization process predominantly resulting in esterification and oligomer formation, with ineffective transesterification. Moreover, the decarboxylation reaction, which terminates polymerization, contributes to the formation of low-molecular-weight products, as mentioned above. Additionally, branching, such as the presence of glycerol, may cause inaccuracies in GPC measurements, as branched polymers occupy a smaller hydrodynamic volume compared to linear polymers of the same molecular weight. Notably, higher dispersity values were observed for PGLI and PGL_80_M_20_I, which may be due to incomplete conversion or uneven chain growth at a lower MA content. As the MA content increases, the polymerization appears more controlled—possibly because MA’s symmetric structure and closely spaced carboxylic groups promote faster and more uniform esterification compared to LA and IA. This reduces the likelihood of branching and side reactions, thereby narrowing the molecular weight distribution. Studies in the literature suggest that these low molecular weights are suitable for obtaining cross-linked polymer films.^25,26^ Thus, in this study, these oligomers were used in the cross-linking (curing) step. Scheme 2 illustrates the dual cross-linking process of PGLMI oligomers. The first mechanism involves free-radical cross-linking initiated by dicumyl peroxide (DCP), which decomposes thermally to generate radicals that abstract hydrogen atoms from allylic positions in the itaconate units, leading to carbon-centered radicals and subsequent covalent bond formation (Scheme S3). The second mechanism proceeds through thermal esterification between hydroxyl groups and unreacted carboxylic acid groups in malonic acid. This combined approach—referred to here as dual cross-linking—enhances the network density and contributes to the thermomechanical performance of the resulting films.

**Scheme 2:**
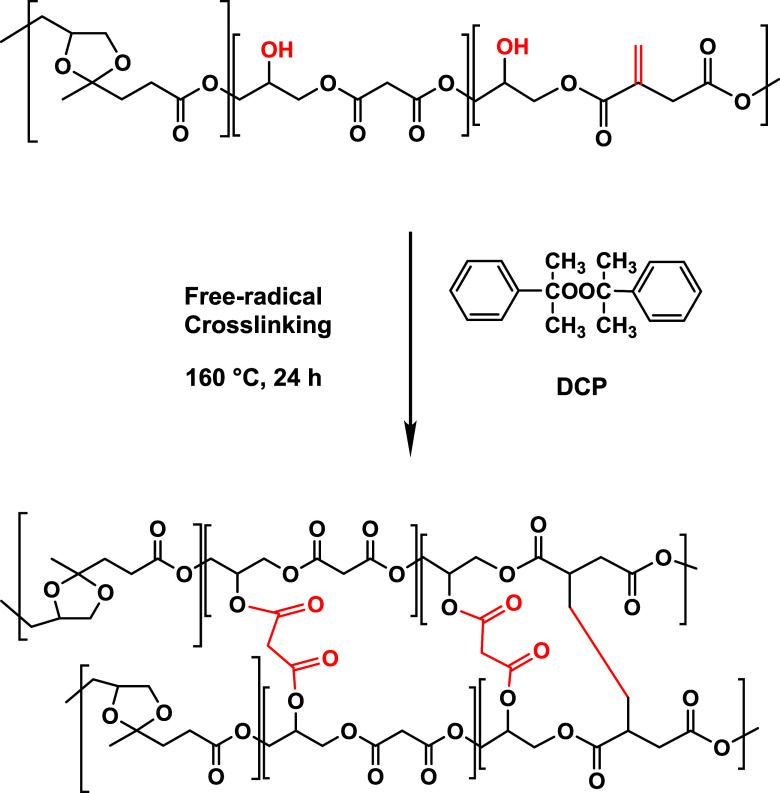
Dual Cross-Linking Mechanism of PGLMI Oligomers[Fn s2fn2]

a (1) Free-radical initiation by dicumyl peroxide (DCP), generating radicals that abstract hydrogen atoms from itaconate units, forming carbon-centered radicals, which covalently link polymer chains; (2) thermal esterification between hydroxyl groups and residual carboxylic acid groups of malonic acid (MA), resulting in additional covalent cross-links.

#### FTIR Spectroscopy

FTIR spectra were taken of the synthesized PGLMI oligomers and cross-linked films to evaluate chemical composition, and they are shown in Figures 2, S2, and S3. A broad O–H stretch peak can be seen around 3400 cm^–1^, indicating a −OH of glycerol-terminal groups. Peaks around 2900 cm^–1^ correspond to the C–H stretching. A strong absorption peak at approximately 1715 cm^–1^ is attributed to the C=O stretching vibrations of the ester group. Another characteristic peak with a small shoulder around 1646 cm^–1^ assigned to the C=C stretching from itaconic acid was hardly observed due to overlapping with the peak at 1715 cm^–1^.

**Figure 2:**
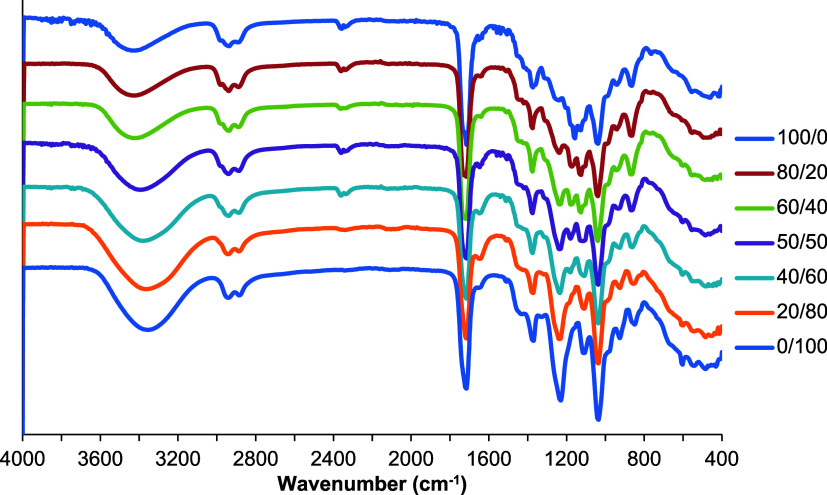
FTIR spectra of uncured PGLMI oligomers.

A direct comparison of FTIR spectra of uncured oligomers with varied GL and GM compositions shows that the peaks of the O–H stretching at 3400 cm^–1^ systematically and proportionally shift to lower frequency but higher intensity with the increase of GM in the composition. Since GM segments have more hydroxyl groups on the chains and ends, the varied structure of GL and GM results in a shift in peaks. Moreover, the symmetric C–O stretching peak at 1221 cm^–1^ is obvious in PGMI, and the intensity of the peak proportionally increases with the decrease in the GL content, which agrees well with the copolyester compositions. The peak at 1125 cm^–1^ assigned to the C–O–C stretching in the 1,3-dioxolane-type ketal group of GL and ester bonds shifted to lower frequency with the increase in the malonate content in the copolyesters.

The FTIR spectra of PGLMI before and after curing are shown in Figure 3. The presence of cross-linking is confirmed by the decrease in intensity of the characteristic C=C stretching bands at 1646, 928, and 863 cm^–1^ (C=C deformation).^27^ Moreover, after cross-linking, the intensity of the peak at 3400 cm^–1^, corresponding to the secondary hydroxyl group of glycerol, decreased significantly, indicating another reaction occurring in the polymer structure. As mentioned in the Introduction section, malonic acid (MA) functions both as a monomer and as a cross-linker. We hypothesize that malonic acid acts as a secondary cross-linker, and increasing the amount of MA facilitates cross-linking by forming more cross-links between the polyester chains (Scheme 2).

**Figure 3:**
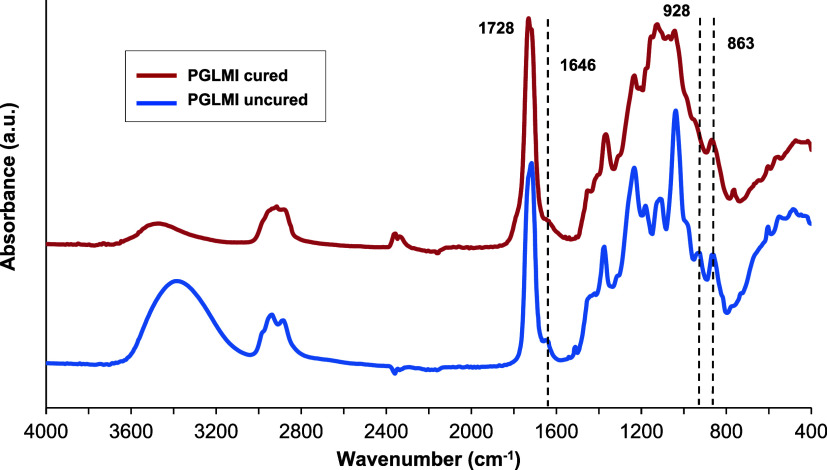
FTIR spectra of cured and uncured PGL_50_M_50_I copolyesters.

### Thermal Properties of Cross-Linked Copolyester Films

#### Thermogravimetric Analysis (TGA)

The thermal properties of cross-linked PGLMI copolyesters and homopolyesters PGLI and PGMI are summarized in Table 2, with TGA and derivative (DTG) curves shown in Figure 4. All synthesized polyesters exhibited thermal degradation temperatures (*T*_max_) in the range of 362–381 °C, which indicates good thermal stability relative to typical requirements for biomedical polymers. For context, biomedical implants often require stability well above sterilization conditions (up to 150 °C).^28^ The temperature at a 5% weight loss (*T*_5%_) varies more significantly with composition, increasing from 154 °C for neat PGMI to 255 °C for PGL_50_M_50_I. This early onset of degradation (*T*_5%_) reflects the initial weight loss that may include residual solvent evaporation or minor polymer chain scission. In particular, the low *T*_5%_ observed for neat PGMI is likely due to the residual solvent absorbed during the synthesis, as supported by solvent removal procedures and TGA trends. The residual solvent presence is not ideal for biomedical use and can limit the long-term thermal stability. The *T*_max_ values indicate the temperature of maximum degradation rate, typically associated with a major polymer chain breakdown. *T*_max_ trends generally follow *T*_5%_ values, with copolyesters showing enhanced thermal stability (up to 381 °C) compared with neat polymers. The improved stability correlates with an increased cross-linking density, which restricts polymer chain mobility and elevates both *T*_max_ and *T*_g_, enhancing resistance to thermal degradation. Despite these favorable *T*_max_ values, the significant difference between *T*_5%_ and *T*_max_ suggests that the initial degradation begins well before maximum polymer breakdown, a factor that must be considered for long-term biomedical applications. Further process optimization is needed to reduce the residual solvent content and improve the onset thermal degradation temperatures (*T*_5%_). Future work will focus on refining purification protocols and cross-linking strategies to enhance the thermal stability and biocompatibility of these materials. At the end, a small amount of ashes (2–9 wt %) was recovered at 600 °C. A single-step degradation behavior can be observed for PGLMI copolyesters, with the degradation primarily attributed to the breakdown of the polymer chains, indicating a major degradation process involving hydrogen bond scission within the polyester structure.^29^ With the ratio of monomers in the copolyesters changed, the degradation temperature was affected, and the maximum decomposition rate reached around 400 °C.

**Figure 4:**
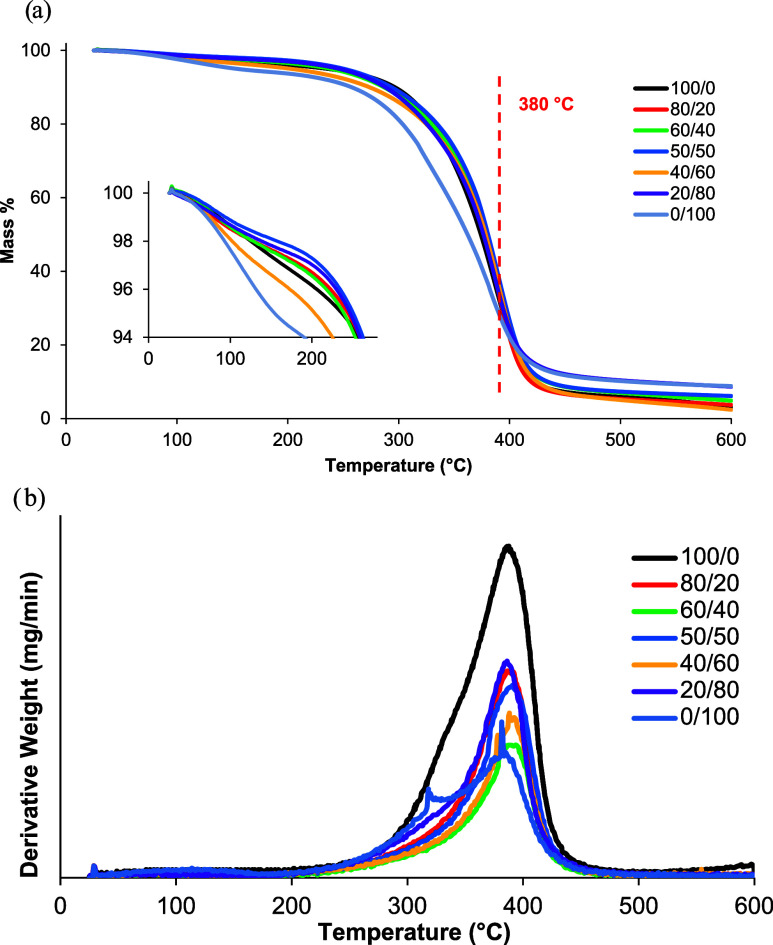
(a) TGA and (b) DTG curves of cured PGLMI copolyesters.

**Table 2:** Thermal Properties of Cured PGLMI Copolyesters^a^

sample	*T*_5%_ (°C)	*T*_max_ (°C)	residue at 600 °C wt %	*T*_g_ (°C)
PGLI	237	374	3.07	–16.2
PGL_80_M_20_I	244	377	3.65	–12.1
PGL_60_M_40_I	239	380	4.85	–7.4
PGL_50_M_50_I	255	381	6.11	–5.5
PGL_40_M_60_I	203	379	2.40	–5.6
PGL_20_M_80_I	247	376	8.66	6.6
PGMI	154	362	8.80	21.3

a *T*_g_, glass-transition temperature; *T*_5%_, temperature at a 5% weight loss; *T*_max_, temperature at a 50% weight loss.

#### Differential Scanning Calorimetry (DSC)

Differential scanning calorimetry (DSC) was utilized to explore the influence of the monomer ratio on the glass-transition temperature (*T*_g_). Figure 5 illustrates the DSC curves of cross-linked neat PGLI, neat PGMI, and PGLMI copolyesters containing various monomer ratios, with their thermograph values summarized in Table 2. Neat PGLI (100/0) and neat PGMI (0/100) exhibited a glass-transition temperature of 21.3 °C and −16.2 °C, respectively. For all of the PGLMI copolyesters, only one Tg between those of neat PGLI and PGMI was observed, which gradually decreases with the increase of MA in the copolyesters and approaches the *T*_g_ of neat PGMI. This phenomenon suggests the formation of a single amorphous phase in all of the PGLMI copolyesters, without any microscale or nanoscale separation.

**Figure 5:**
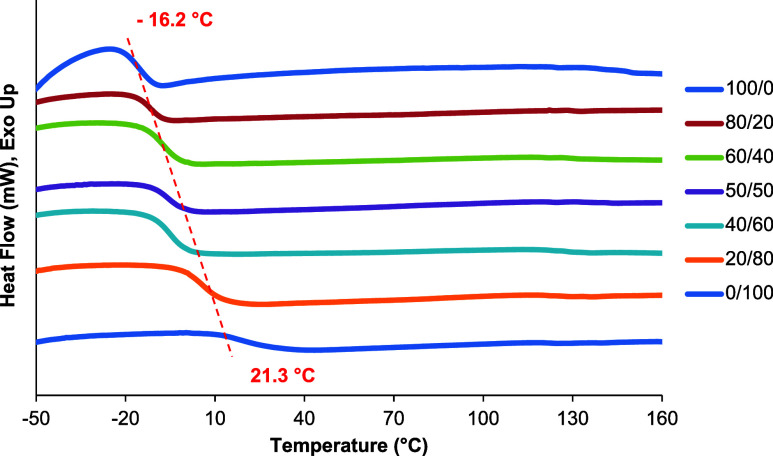
Second heating DSC curves of cured PGLMI copolyesters.

The decrease of the diacid length from LA to MA increases the ester linkage density, which led to a higher amount of intra- or interchain interactions between ester groups, hence the decrease of chain mobility leading to the increase of *T*_g_. For random copolyesters, the variation of *T*_g_ with the composition often follows the Fox equation,^30^ defined by eq 2

2 $$\frac{1}{T_{g,\mathrm{co}}}=\frac{w_{1}}{T_{g,1}}+\frac{w_{2}}{T_{g,2}}$$

where *T*_g,1_ and *T*_g,2_ are the glass-transition temperatures of homopolyesters, and w_1_ and *w*_2_ are their respective mass fractions. As shown in Figure 6, the Fox equation did not adequately fit our experimental data for PGLMI, which appears to contradict the findings of Papageorgiou and Bikiaris.^31^ However, in some cases, the Gordon–Taylor equation,^30^ provided a better description of the Tg variation in random aliphatic copolyesters like PGLMI,^32,33^ as defined by eq 3

3 $$T_{g,\mathrm{co}}=\frac{w_{1}T_{g,1}+k\left(1-w_{1}\right)T_{g,2}}{w_{1}+k\left(1-w_{1}\right)}$$

where *T*_g,1_ and *T*_g,2_ are the glass-transition temperatures of homopolyesters, w_1_ is the respective mass fraction of the homopolyester 1, and *k* is the Gordon–Taylor parameter. As shown in Figure 6, the Gordon–Taylor equation fits our experimental data for PGLMI with a constant of *k* = 0.45. Usually, k is considered as a fitting parameter,^34^ even if in the original version of volume additivity, the parameter *k* = ρ_l_Δα_2_/ρ_2_Δα_1_ was well-defined (ρ_i_ is the density and Δα*_i_* = α_melt_ – α_glass_ is the increment at *T*_g_ of the expansion coefficient of the respective component (*i*)).

**Figure 6:**
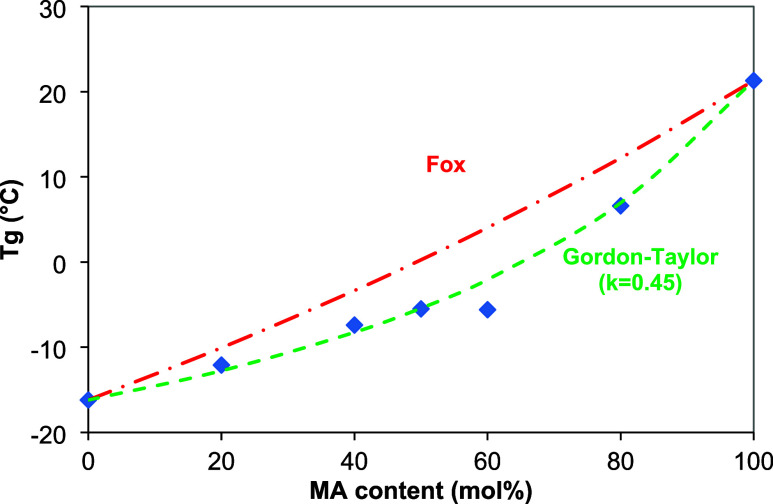
Variation of *T*_g_ vs MA content in cured PGLMI and plots of theoretical Fox and Gordon–Taylor relations.

Both the Fox and Gordon–Taylor equations are commonly used to predict the glass-transition temperature (*T*_g_) of polymer blends or copolymers, but their assumptions may not fully align with the behavior of cross-linked systems. The Fox equation, typically applied to non-cross-linked, random, and homogeneous polymers, did not provide a satisfactory fit to our experimental data. This is likely due to the cross-linked nature of the PGLMI copolyesters, which restricts the chain mobility and alters the thermal behavior, causing the results to deviate from the predictions of the Fox model. On the other hand, the Gordon–Taylor equation, despite also assuming a non-cross-linked system, provided a good fit for our data with a constant *k* = 0.45, suggesting that the interaction between the polymer components (e.g., ester linkages, levulinate units, and malonate units) behaves in a manner that the model can still approximate. The *k* value of 0.45 indicates a moderate interaction between the polymer segments, supporting the applicability of the Gordon–Taylor model for this system. While cross-linking typically reduces chain mobility and can influence the Tg, the degree of cross-linking in this case does not appear to significantly impact the predictions of the Gordon–Taylor equation, making it a reasonable approximation for the PGLMI copolyesters.

#### DMA

As the levulinic acid content decreases, the film products shift from soft to rigid form. This change is believed to be due to malonic acid acting as a secondary cross-linker, which also alters the surface morphology (Scheme 2). Dynamic mechanical analysis (DMA) was used to determine the thermomechanical properties of the cured copolyesters. It was conducted only for the PGMI copolyester due to its suitability for testing. The other samples were found to be unsuitable for analysis because of challenges related to their sticky nature. Figure S4 presents the DMA curves for the storage modulus (*E*′), loss modulus (*E*″), and tan δ as a function of temperature for the PGMI polyesters. The onset of the storage modulus decay occurred at approximately −16.9 °C, marking the beginning of the glass-transition region. The main α-relaxation, corresponding to the tan δ peak, was observed at 21.7 °C, which aligns well with the *T*_g_ obtained by DSC. Notably, a weak shoulder appears near 10 °C, suggesting a possible secondary relaxation or heterogeneity in the polymer network. This could arise from microphase separation or regions of different cross-link densities, though further investigation would be required to confirm this. The transition was observed with increasing temperature between −50 and 50 °C, as the storage and loss moduli decreased. The *E*′ value was 725 MPa at −40 °C, while it was 3.8 MPa at 30 °C.

The cross-link density (*v*_e_) was determined based on the kinetic theory of rubber elasticity by using the following equation^35^

4 $$v_{e}=\frac{E^{\prime }}{3\mathrm{RT}}$$

where *R* is the gas constant (8.314 J/molK), *T* is the absolute temperature in *K*, and the flexural storage modulus (*E*′) is obtained in the rubbery plateau. Herein, the onset of the rubbery plateau in the storage modulus was used to estimate the cross-link density. Based on this approach, the cross-link density was determined as 0.265 × 10^–3^ mol/cm^3^. This method assumes an ideal network in which all chains contribute effectively to elastic deformation.

### Shape Memory Properties

A cross-linked PGL_20_M_80_I copolyester was used to demonstrate the shape memory feature, which is another property of the cross-linked LA-based copolyesters (Figure 7). The sample in the form of a film strip was bent above the *T*_g_ value, and this shape was fixed at a temperature below *T*_g_. Later, when the sample was left at room temperature again, it was observed that it reached a permanent shape with a 99% recovery ratio in a very short time. Shape memory properties of the other copolyesters could not be determined quantitatively due to their sticky nature during bending. Movie S1 shows a visual representation of how the cross-linked PGL_20_M_80_I copolyester film behaves after being placed in a freezer at −20 °C.

**Figure 7:**
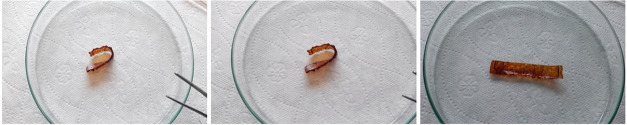
Representative shape memory performance of cured PGL_20_M_80_I copolyester samples at room temperature.

### Swelling Properties

The cross-linking density of the cured PGMI and PGL_20_M_80_I copolyesters was investigated by determining the gel content within the cross-linked polymers. PGL_20_M_80_I was chosen as a representative copolyester to explore the effects of the monomer ratio. The gel content of PGMI and PGL_20_M_80_I was measured by swelling the cross-linked polymers in THF (Table S1). The gel content was retained, and the sol content was eliminated. It was found that the gel content of PGMI was about 96.14%, which is higher than that of PGL_20_M_80_I, indicating that the PGMI cured for 24 h has a higher cross-linking density compared to PGL_20_M_80_I. This is consistent with the calculations of the swelling ratio and the cross-linking density. Moreover, the molar mass Mc of the cross-linked polymers was calculated (SI), and for the PGMI, it is lower than that for the PGL_20_M_80_I, which is less cross-linked. The water uptake properties of PGMI and PGL_20_M_80_I were investigated by swelling measurements of the polymers in distilled water at room temperature. The results showed that the water content absorbed by PGMI was much higher than that of PGL_20_M_80_I. The water uptake ratio of PGMI decreases with the incorporation of LA.

### In Vitro Degradation

Figure 8a illustrates the variation in weight loss of the cross-linked copolyester films produced using different levulinic acid/malonic acid ratios in PBS over an 8-week period. The degradation properties of polymers are influenced by their chemical structure. Polyester such as PGLMI can be degraded under physiological conditions because the ester bond is a hydrolyzable chemical bond^36^ Following a 7-day incubation time, the structural integrity of the films was significantly impaired with a noticeable decrease in weight and according to the percentage remaining by mass, 50/50 (59.49 ± 12.32%), 60/40 (40.75 ± 14.49%), 80/20 (37.89 ± 4.60%), and 100/0 (47.29 ± 0%). It was established that 100/0 and 80/20 exhibited total loss of their film structures by the conclusion of the fourth week, while 60/40 demonstrated this outcome by the end of the fifth week. Subsequent to the experimental period, it was observed that the film structures comprising the 20/80 and 40/60 ratios exhibited a capacity for retaining their original configuration. The remaining mass percentage of each respective sample was recorded as 20/80 (48.44 ± 6.39%) and 40/60 (37.18 ± 0%). The results showed that the degradation rate of PGLMI decreased with an increase of the MA ratio in PGLMI copolyesters. In contrast, neat PGMI degraded faster than PGLMI copolyesters after 8 weeks. As the MA ratio increases in the copolyesters, the presence of ester bonds becomes more prominent. These ester groups are susceptible to hydrolytic degradation in aqueous environments like PBS. However, at higher MA ratios, the cross-linking density may also increase, which can limit water uptake and restrict the exposure of ester bonds to water. This could lead to slower in vitro degradation compared to lower MA-containing samples, where the polymer is more easily hydrated and degraded. The shape and degradation behavior of the samples suggested that bulk erosion may take place, and they became soft and sticky after soaking in PBS for several weeks. Furthermore, the degradation study was carried out until the films became too sticky to be taken out completely.

**Figure 8:**
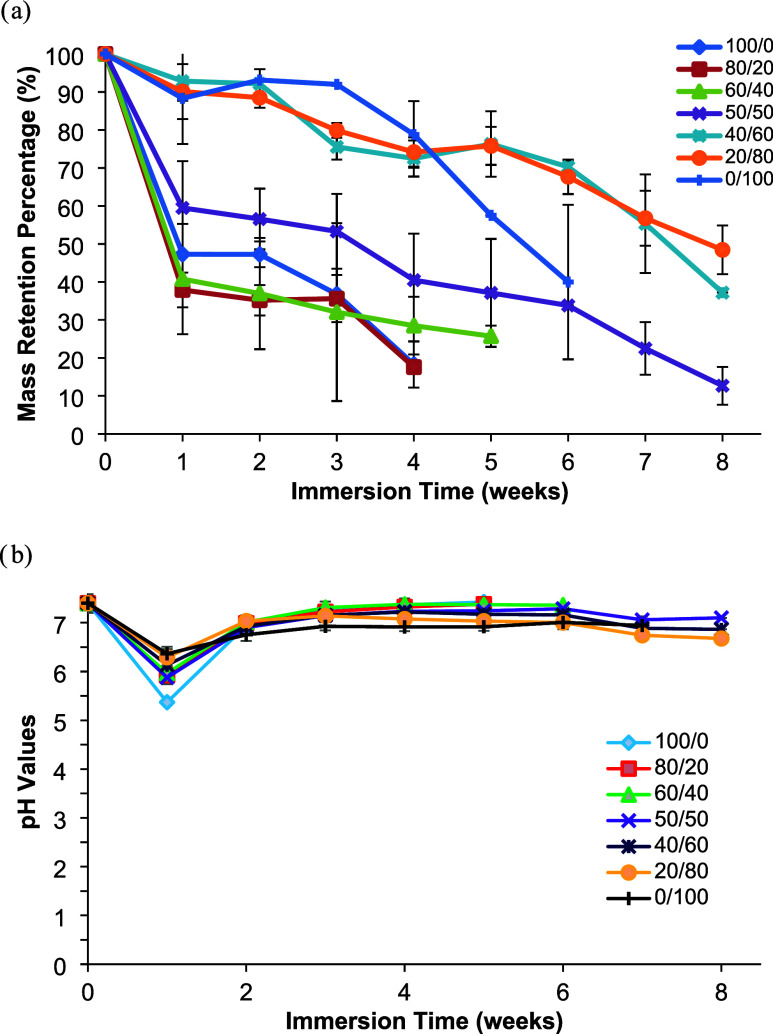
(a) Mass retention of cross-linked PGLMI random copolyesters with immersion time in PBS (*n* = 3). (b) The change of pH of the immersion fluids (*n* = 3). The bars represent mean ± standard deviation.

Several commercial biodegradable polymer stents, such as Synergy, Orsiro, Yukon Choice Flex, Ultimaster, and Biomatrix, offer varying degradation times and drug release profiles to aid coronary healing.^37–40^ The degradation times for these stents range from 3 to 24 months, with drug release occurring within 1–6 months. These stents use different biodegradable polymers like PDLLA, PLA, and PLGA. For example, the Synergy stent degrades in 4 months, Orsiro in 12–24 months, Yukon Choice Flex in 6–9 months, Ultimaster in 3–4 months, and Biomatrix in 6–9 months. The degradation profile of synthesized PGL_20_M_80_I (20/80) copolyesters, with a 48.44% remaining mass after 8 weeks, is comparable to that of these stents.

The pH variation of the soaking solution was also tested due to the acidic degradation products. At the end of the first week, as shown in Figure 8b, there was a significant decrease in the immersion solution of the complete set as a result of diffusion of unreacted acidic monomers and degradation products. After 7 days of incubation, the pH values of the immersion solutions were less than 7.0 (100/0 (pH 5.37 ± 0.18), 80/20 (pH 5.92 ± 0.18), 60/40 (pH 5.97 ± 0.20), 50/50 (pH 5.88 ± 0.09), 40/60 (6.13 ± 0.03), 20/80 (pH 6.29 ± 0.17), and 100/0 (pH 6.36 ± 0.14)). However, the pH variation of the immersion solutions was consistent with the trend of the mass loss. Namely, the pH values observed after the first week were similar and remained around 7.0, indicating a linear degradation process.

## Conclusions

Fully biobased oligomers were successfully synthesized from glycerol, levulinic acid (LA), malonic acid (MA), and itaconic acid (IA) via melt polycondensation. The monomer molar ratio was found to significantly enhance the thermal stability of the random copolyester films, which were cross-linked through two complementary mechanisms: radical initiation by dicumyl peroxide (DCP) and covalent cross-linking by MA. In particular, PGLMI copolyesters with a 50/50 molar ratio exhibited high thermal degradation temperatures (255 and 381 °C), making them suitable for applications requiring resistance to elevated temperatures. The glass-transition temperature (*T*_g_) could be tuned by varying the MA content, reaching room temperature, and providing versatility for applications where thermal responsiveness is crucial. All PGLMI copolyesters exhibited a single *T*_g_, which increased with a higher MA content. Additionally, the cross-linked copolyesters demonstrated a promising shape memory effect that could be adjusted through composition, making them ideal for stent applications, where both shape recovery at body temperature and controlled degradation are critical for optimal performance. Overall, these biobased copolyesters offer a favorable combination of thermal stability, mechanical properties, shape memory behavior, and controlled degradation, making them strong candidates for implantable medical devices.

## Supplementary Material





## Notes

Mechanistic schemes; Fischer esterification; intramolecular ketalization; decarboxylation; free-radical initiation by DCP; swelling test methodology; cross-linking analysis equations (Flory–Rehner); NMR spectra (^1^H, ^13^C) of uncured PGLMI oligomers; FTIR spectra of uncured and cured PGLMI copolyesters; DMA curves of cured films; and swelling property table of cross-linked copolyester films (PDF)
A 20/80 film sample (AVI)

## References

[1] Dirauf M., Muljajew I., Weber C., Schubert U. S. (2022). Recent advances
in degradable synthetic polymers for biomedical applications-Beyond
polyesters. Prog. Polym. Sci..

[2] Moshkbid E., Cree D. E., Bradford L., Zhang W. (2024). Biodegradable alternatives
to plastic in medical equipment: current state, challenges, and the
future. J. Compos. Sci..

[3] Mayakrishnan, V. ; Murugan, P. A. Degradation Studies of Resorbable Materials for Biomedical Applications. In Nanomanufacturing Techniques in Sustainable Healthcare Applications; CRC Press, 2024; pp 258–277.

[4] Pellis A., Malinconico M., Guarneri A., Gardossi L. (2021). Renewable
polymers
and plastics: Performance beyond the green, New. Biotechnology.

[5] Iwata T. (2015). Biodegradable
and Bio-Based Polymers: Future Prospects of Eco-Friendly Plastics. Angew. Chem., Int. Ed..

[6] Rosenboom J.-G., Langer R., Traverso G. (2022). Bioplastics
for a circular economy,
Nature Reviews. Materials.

[7] Garrison T. F., Murawski A., Quirino R. L. (2016). Bio-based
polymers with potential
for biodegradability. Polymers.

[8] Fujieda K., Enomoto Y., Zhang Y., Iwata T. (2022). Synthesis and characterization
of novel potentially biodegradable aromatic polyesters consisting
of divanillic acids with free phenolic hydroxyl groups. Polymer.

[9] Kabe T., Okumura S., Gan H., Ilangovan M., Iwata T. (2024). Thermal properties and crystallization behavior of Curdlan acetate
propionate mixed esters. Macromolecules.

[10] Werpy, T. ; Petersen, G. ; Aden, A. ; Bozell, J. ; Holladay, J. ; White, J. ; Manheim, A. ; Eliot, D. ; Lasure, L. ; Jones, S. Top Value Added Chemicals From Biomass. Vol. 1-Results of Screening for Potential Candidates From Sugars and Synthesis Gas, DTIC Document 2004.

[11] Isikgor F. H., Becer C. R. (2015). Lignocellulosic
biomass: a sustainable platform for
the production of bio-based chemicals and polymers. Polymer chemistry.

[12] Godinho B., Nogueira R., Gama N., Ferreira A. (2023). Synthesis of prepolymers
of poly (glycerol-co-diacids) based on sebacic and succinic acid mixtures. ACS omega.

[13] Hayes G. C., Becer C. R. (2020). Levulinic acid:
a sustainable platform chemical for
novel polymer architectures. Polym. Chem..

[14] Zhang Y., Wu L., Li F., Li B. (2006). Lactonization and condensation polymerization
of 5-hydroxylevulinic acid. J. Chem. Ind. Eng.
China.

[15] Yan Z., Zhenghong G., Jie C., Zhengping F. (2009). Synthesis
and characterization of biodegradable poly (5-hydroxylevulinic ACID-co-L-lactic
acid). Acta Polym. Sin..

[16] Amarasekara A. S., Animashaun M. A. (2016). Acid catalyzed competitive esterification
and ketalization
of levulinic acid with 1, 2 and 1, 3-diols: the effect of heterogeneous
and homogeneous catalysts. Catal. Lett..

[17] Amarasekara A. S., Hawkins S. A. (2011). Synthesis of levulinic
acid–glycerol ketal–ester
oligomers and structural characterization using NMR spectroscopy. Eur. Polym. J..

[18] Amarasekara A. S., Ha U., Okorie N. C. (2018). Renewable
polymers: Synthesis and characterization
of poly (levulinic acid-pentaerythritol). J.
Polym. Sci. Part A.

[19] Mitra T., Sailakshmi G., Gnanamani A., Mandal A. (2012). Preparation and characterization
of malonic acid cross-linked chitosan and collagen 3D scaffolds: an
approach on non-covalent interactions. J. Mater.
Sci.: Mater. Med..

[20] Zhao W., Nolan B., Bermudez H., Hsu S. L., Choudhary U., van Walsem J. (2020). Spectroscopic study of the morphology
development of
closed-cell polyurethane foam using bio-based malonic acid as chain
extender. Polymer.

[21] Cadar O., Paul M., Roman C., Miclean M., Majdik C. (2012). Biodegradation
behaviour of poly (lactic acid) and (lactic acid-ethylene glycol-malonic
or succinic acid) copolymers under controlled composting conditions
in a laboratory test system. Polym. Degrad.
Stab..

[22] Kareem Z., Gulec H. R., Karaboyun M., Susgun S., Eyiler E. (2025). Synthesis
of Levulinic Acid-Based Block Copolyesters with Tunable Degradation. ACS Appl. Polym. Mater..

[23] Tan B., Bi S., Emery K., Sobkowicz M. J. (2017). Bio-based poly (butylene succinate-co-hexamethylene
succinate) copolyesters with tunable thermal and mechanical properties. Eur. Polym. J..

[24] Guo B., Chen Y., Lei Y., Zhang L., Zhou W. Y., Rabie A. B. M., Zhao J. (2011). Biobased Poly­(propylene
sebacate)
as Shape Memory Polymer with Tunable Switching Temperature for Potential
Biomedical Applications. Biomacromolecules.

[25] Lok T.-J., Wong J.-W., Li X., Fu Y., Xue Y., Jamaludin F. H., Fong M., Edward E. B., Ma C., Chandren S. (2024). Biobased Itaconate Polyester Thermoset
with
Tunable Mechanical Properties. Macromolecules.

[26] Ramirez-Suarez J. H., Käfer F., Ober C. K., Goddard J. M. (2024). Synthesis of Itaconate-Based
Biopolyesters with Improved Polycondensation Control. ACS Appl. Polym. Mater..

[27] Melilli G., Guigo N., Robert T., Sbirrazzuoli N. (2022). Radical oxidation
of itaconic acid-derived unsaturated polyesters under thermal curing
conditions. Macromolecules.

[28] Herczeg C. K., Song J. (2022). Sterilization of polymeric
implants: challenges and opportunities. ACS
Appl. Bio Mater..

[29] Laurichesse S., Huillet C., Avérous L. (2014). Original polyols
based on organosolv
lignin and fatty acids: new bio-based building blocks for segmented
polyurethane synthesis. Green Chem..

[30] Debuissy T., Pollet E., Avérous L. (2017). Synthesis
and characterization of
biobased poly (butylene succinate-ran-butylene adipate). Analysis
of the composition-dependent physicochemical properties. Eur. Polym. J..

[31] Papageorgiou G. Z., Bikiaris D. N. (2007). Synthesis, cocrystallization,
and enzymatic degradation
of novel poly (butylene-co-propylene succinate) copolymers. Biomacromolecules.

[32] Debuissy T., Pollet E., Avérous L. (2017). Synthesis
and characterization of
fully biobased poly (propylene succinate-ran-propylene adipate). Analysis
of the architecture-dependent physicochemical behavior. J. Polym. Sci., Part A: Polym. Chem..

[33] Safari M., de Ilarduya A. M., Mugica A., Zubitur M., Muñoz-Guerra S., Müller A. J. (2018). Tuning the thermal properties and morphology of isodimorphic
poly [(butylene succinate)-ran-(ε-caprolactone)] copolyesters
by changing composition, molecular weight, and thermal history. Macromolecules.

[34] Penzel E., Rieger J., Schneider H. (1997). The glass
transition temperature
of random copolymers: 1. Experimental data and the Gordon-Taylor equation. Polymer.

[35] Fidanovski B. Z., Spasojevic P. M., Panic V. V., Seslija S. I., Spasojevic J. P., Popovic I. G. (2018). Synthesis and characterization of
fully bio-based unsaturated
polyester resins. J. Mater. Sci..

[36] Jia Y., Wang W., Zhou X., Nie W., Chen L., He C. (2016). Synthesis
and characterization of poly (glycerol sebacate)-based
elastomeric copolyesters for tissue engineering applications. Polym. Chem..

[37] Hassan S., Ali M. N., Ghafoor B. (2022). Evolutionary
perspective of drug
eluting stents: from thick polymer to polymer free approach. J. Cardiothorac. Surg..

[38] Rebagay G., Bangalore S. (2019). Biodegradable polymers and stents:
the next generation?. Curr. Cardiovasc. Risk
Rep..

[39] Hou D., Huibregtse B., Dawkins K., Donnelly J., Roy K., Chen J. P., Akinapelli A. (2017). Current state of bioabsorbable polymer-coated
drug-eluting stents. Curr. Cardiol. Rev..

[40] Hu T., Yang C., Lin S., Yu Q., Wang G. (2018). Biodegradable
stents for coronary artery disease treatment: Recent advances and
future perspectives. Mater. Sci. Eng.: C.

