# Supplementary material for: Development of Biodegradable and Biobased Poly(glycerol levulinate-co-glycerol malonate) Copolyesters with Controlled Degradation
Source: Macromolecules. 2025 Sep 10;58(18):9952–61. doi: 10.1021/acs.macromol.5c00383 (PMC12461924; doi:10.1021/acs.macromol.5c00383)
Supplement: Supplementary file 1 [file ma5c00383_si_001.pdf]

Development of Biodegradable and Biobased Poly(glycerol levulinate-co-glycerol malonate) Copolyesters with Controlled Degradation

*Huru Rabia Gulec, Zaid Kareem, Mete Karaboyun, and Ersan Eyiler**

Department of Chemical Engineering, Cukurova University, Adana 01950, Turkey

E-mail: eeyiler@gmail.com

Supporting Information

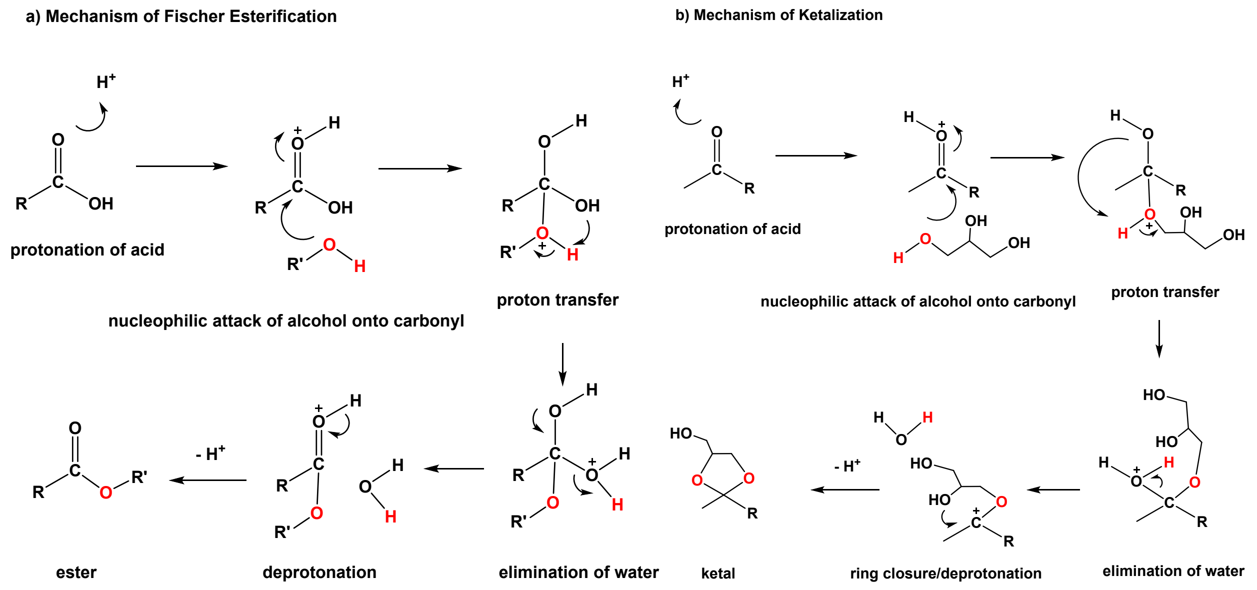

Scheme S1. a) Fischer esterification mechanism between glycerol and carboxylic acids (levulinic, malonic, and itaconic acids), forming polyester linkages under acid-catalyzed melt polycondensation conditions. b) Sb_2O_3-catalyzed intramolecular ketalization of levulinic acid's ketone group with vicinal diols of glycerol, yielding a stable five-membered 1,3-dioxolane ring.

Swelling Test and Crosslinking Analysis

The degree of crosslinking in the synthesized copolyesters was evaluated via swelling experiments in tetrahydrofuran (THF). Crosslink density is a critical factor influencing thermal and mechanical properties; higher crosslink density typically increases hardness, reduces elongation, decreases swelling capacity, and raises the glass transition temperature.

Samples (10 × 5 × 0.6 mm) were first weighed in their dry state (w_i), then immersed in THF for 24 h at room temperature. After swelling, the swollen weight (w_s) was recorded. The samples were then dried under vacuum until constant weight (w_d).

The swelling ratio (Q) was calculated using [1]:

$$Q = 1 + (w_s/w_d - 1) \times (\rho_p/\rho_s) \quad \text{(S1)}$$

where ρ_p is the density of the polymer (measured via the pycnometer method), and ρ_s is the density of THF (0.889 g/cm^3) [2].

The crosslinking density (v_d) of the samples was calculated using the Flory-Rehner equation [2]:

$$v_d = - [\ln(1-Q^{-1}) + Q^{-1} + XQ^{-2}] / [\Phi(Q^{-1/3} - Q^{-1/2})] \quad \text{(S2)}$$

where X is the Flory polymer interaction parameter, and Φ is the molar volume of THF.

The molecular weight between crosslinks (Mc) was obtained using:

$$Mc = \rho_p/v_d \quad \text{(S3)}$$

The gel content (%) was calculated from the weight ratio before and after THF extraction:

$$\text{Gel content (\%)} = \frac{w_d}{w_i} \times 100 \quad \text{(S4)}$$

The water uptake (%) was also assessed by soaking samples in distilled water for 24 h and calculating:

$$\text{Water uptake (\%)} = (w_s - w_d) / w_d \times 100 \quad \text{(S5)}$$

a)

b)

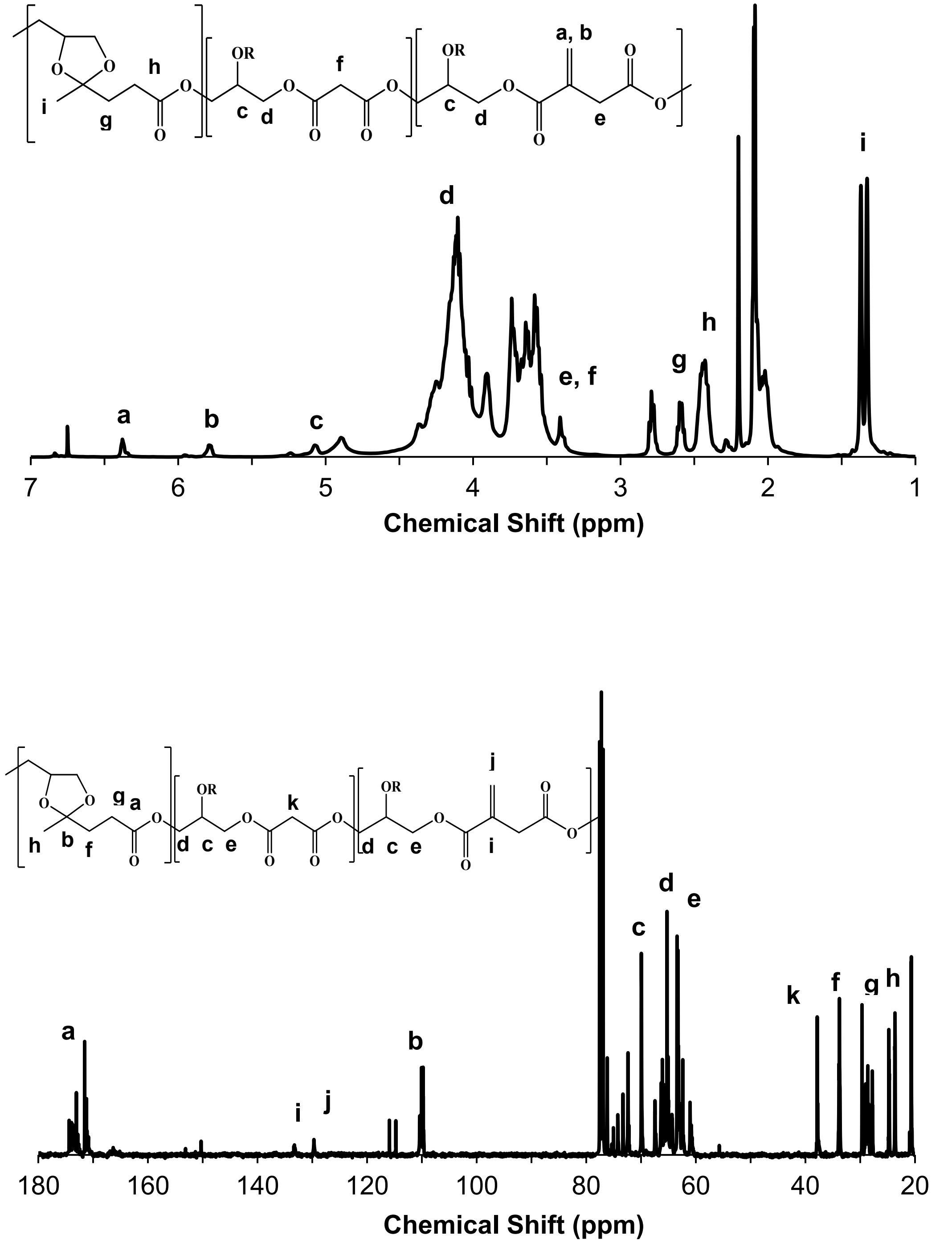

Fig. S1. Representative (a) ^{1}H and (b) ^{13}C NMR spectra for uncured $PGL_{50}M_{50}I$ oligomers.

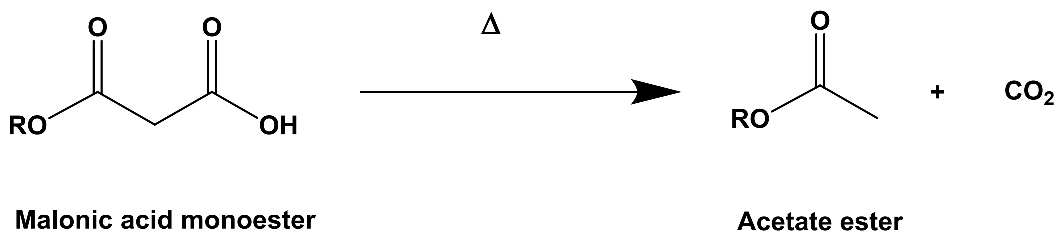

Scheme S2. Decarboxylation mechanism of PGLMI oligomers.

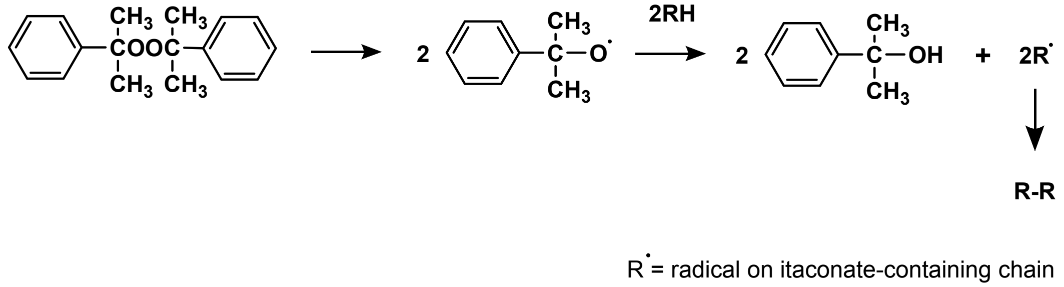

Scheme S3. Free-radical initiation by DCP: thermal cleavage generates radicals that abstract hydrogen from itaconate units, forming polymer radicals that crosslink.

a)

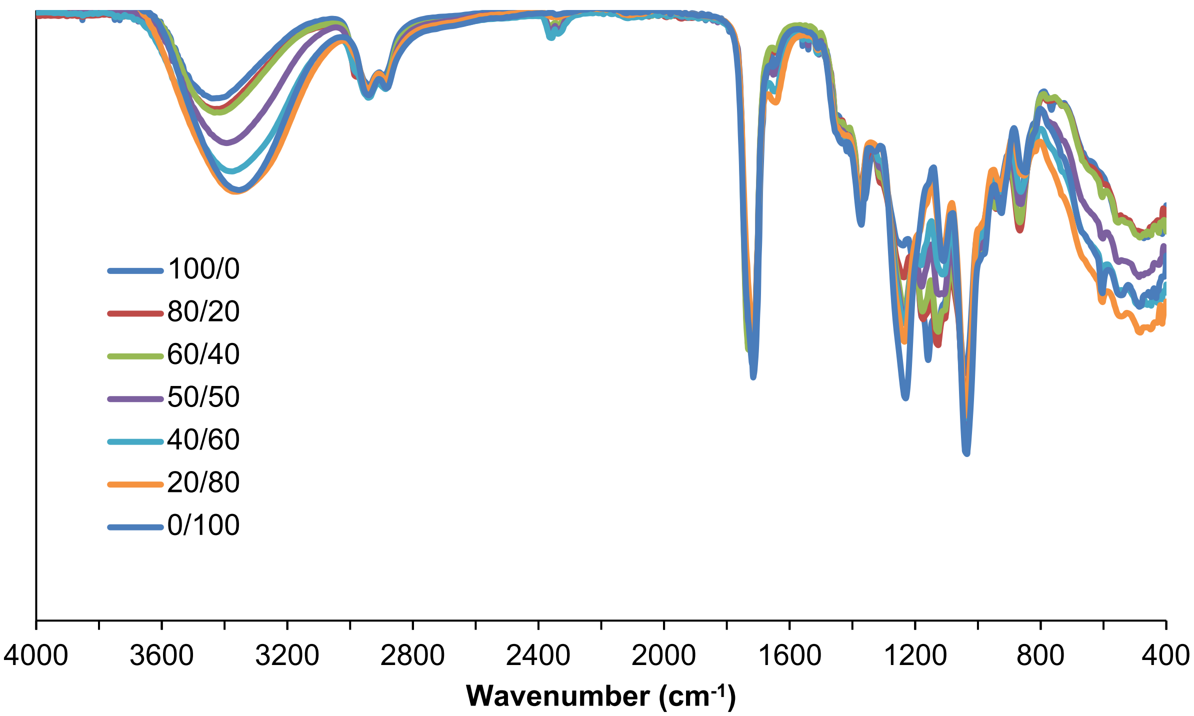

b)

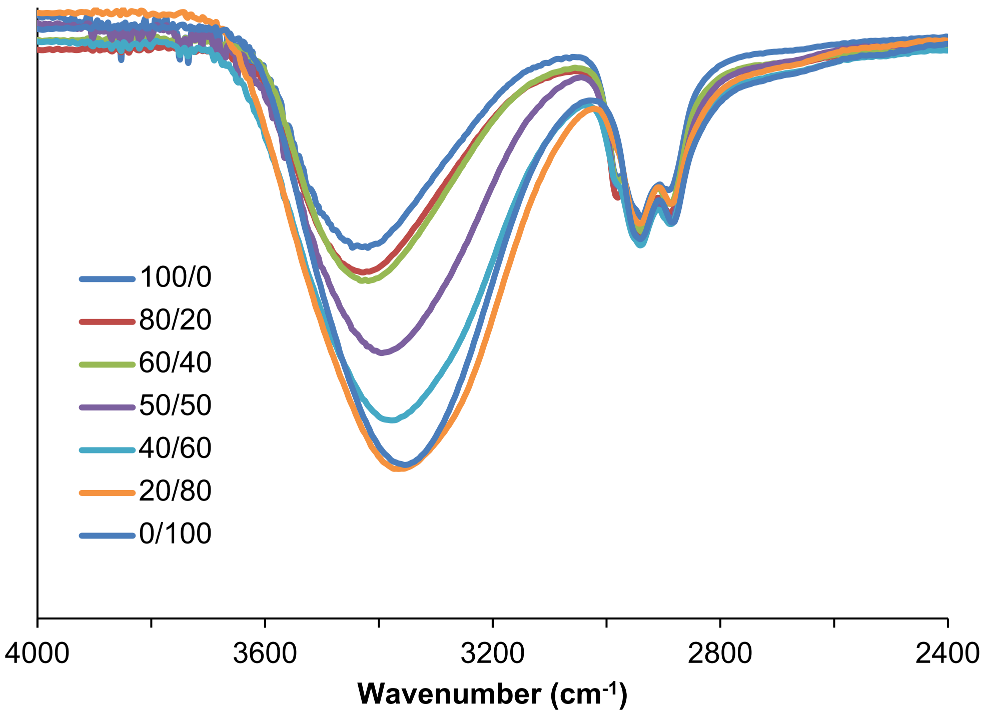

Fig. S2. FTIR spectra of uncured PGLMI oligomers: (a) 400 – 4000 cm^{-1} and (b) 2400 – 4000 cm^{-1}.

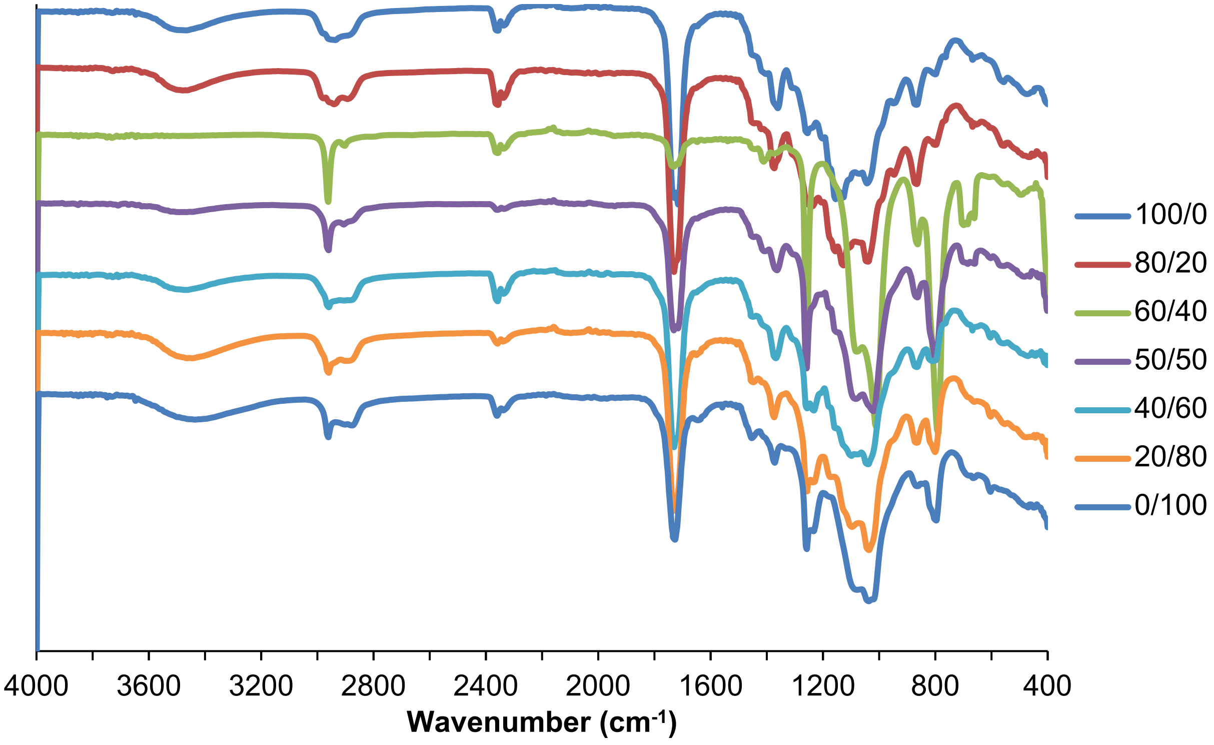

Fig. S3. FTIR spectra of cured PGLMI copolyester films.

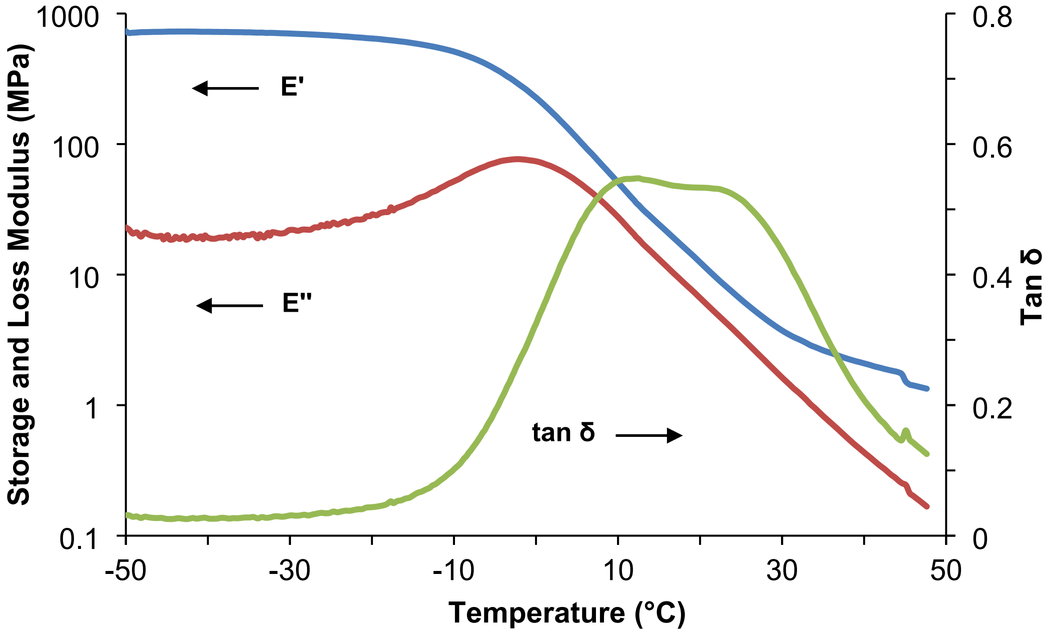

Fig. S4. DMA curves of cured PGMI film sample.

Table S1. Swelling Properties of crosslinked PGLMI copolyester films.

Sample	Gel Content (%)	Swelling Ratio (%, THF)	Water Uptake (%)	Crosslinking Density (mol/cm^3, x10^3)	Mc (g/mol)
$PGL_{20}M_{80}I$	70.37±4.61	58.34±10.23	44.71±3.44	26.11±8.48	42.89±17.06
PGMI	96.14±3.14	32.74±6.02	78.74±7.03	86.63±26.98	12.82±4.84

[1] Y. Jia, W. Wang, X. Zhou, W. Nie, L. Chen, C. He, Synthesis and characterization of poly (glycerol sebacate)-based elastomeric copolyesters for tissue engineering applications, Polymer Chemistry 7(14) (2016) 2553-2564.

[2] A. Kaltenegger-Uray, G. Rieß, T. Lucyshyn, C. Holzer, W. Kern, Physical foaming and crosslinking of polyethylene with modified talcum, Polymers 11(9) (2019) 1472.
